# Association between Physical Activity and Respiratory Diseases in Adolescents: An Age- and Gender-Matched Study

**DOI:** 10.3390/ijerph18041397

**Published:** 2021-02-03

**Authors:** Jeong-Hui Park, Eunhye Yoo, Myong-Won Seo, Hyun Chul Jung, Jung-Min Lee

**Affiliations:** 1 Department of Physical Education, Global Campus, Kyung Hee University, 1732 Deokyoungdaero, Giheung-gu, Yongin-si 17014, Gyeonggi-do, Korea; jeonghee@khu.ac.kr; 2 Department of Physical Education, Seoul National University, 1 Gwanakro, Gwanakgu, Seoul 08826, Korea; yeh04@snu.ac.kr; 3 Department of Taekwondo, Global Campus, Kyung Hee University, 1732 Deokyoungdaero, Giheung-gu, Yongin-si 17014, Gyeonggi-do, Korea; myongwonseo@khu.ac.kr; 4 Department of Coaching, Global Campus, Kyung Hee University, 1732 Deokyoungdaero, Giheung-gu, Yongin-si 17014, Gyeonggi-do, Korea; jhc@khu.ac.kr

**Keywords:** physical activity, body mass index, asthma, allergic rhinitis, adolescents

## Abstract

The purpose of the present cross-sectional study was to examine the impacts of allergic respiratory diseases on physical activity (PA), sedentary behaviors (SB), and body mass index (BMI) by matching age and gender with those adolescents without allergic respiratory diseases. This present study analyzed data from the 2019 Korea Youth Risk Behavior Web-based Survey (KYRBWS). Among 57,303 Korean adolescents who responded to the survey, the study divided adolescents into three different groups (i.e., general, asthma, and allergic rhinitis group). Asthma and allergic rhinitis groups included adolescents who checked on asthma- or allergic rhinitis-related questions as ‘yes’ (n = 259, n = 259), but the general group responded to any diseases-related question as ‘no’ (n = 259). The age and gender of participants among the three groups were matched. The results showed weight and BMI were significantly higher in asthma and allergic rhinitis groups compared to the general group (*p* < 0.001, *p* < 0.001). Furthermore, age, asthma, and allergic rhinitis were observed to be strong risk factors for predicting obesity in adolescents (BMI, >25.0 kg/m^2^). In addition, this study found allergic respiratory diseases strong impacts on BMI levels because adolescents with ongoing asthma, or allergic rhinitis symptoms were more likely to have the inevitability of further weight gain compared to the general adolescents. Therefore, not only national interest in adolescents with allergic respiratory disease is essential, but PA should be encouraged to prevent and alleviate these diseases due to obesity.

## 1. Introduction

In modern society, an increasing number of adolescents are suffering from allergic respiratory diseases because of a combination of environmental and genetic factors such as air pollution, frequent contact with synthetic materials, and changes in diet [1,2]. According to the International Study of Asthma and Allergies in Childhood (ISAAC), the prevalence of adolescents’ allergic respiratory diseases have increased annually over the past decade [3] and this unfavorable pattern will continue to increase to have an additional 400 million patients by 2025 [4,5]. Moreover, asthma and allergic rhinitis are common allergic respiratory diseases and there is a strong correlation between the diseases. Asthma is a chronic lower respiratory disease with symptoms such as hypersensitivity of the airway, phlegmatic cough, and chest compressions, while allergic rhinitis is an upper respiratory disease with symptoms such as itching, runny nose, and sneezing [6]. In particular, asthma and allergic rhinitis are known to the same pathological phenomenon because two different diseases have the very similar inflammatory response and inflammation form, so they commonly have occurred as comorbidities [7,8,9]. Therefore, it has been reported 80% of asthma patients are accompanied by allergic rhinitis [10], and 20–50% of allergic rhinitis patients are known to develop asthma [11]. 

Allergic respiratory diseases are caused by a combination of diverse factors. Overweight and obesity during childhood and adolescence have been considered as one of the substantial factors that making the diseases more severe. Several studies have confirmed the close link between the rate of increased childhood obesity and asthma exacerbations [12,13]. For instance, a previous study reported 35.68% of overweight and 12.53% of obese children were more likely to have an increased prevalence of allergic rhinitis and atopic dermatitis [14], and it is a notable issue as the global prevalence of allergic rhinitis is still increasing while the prevalence of asthma has leveled off in recent years [15,16]. However, these findings have not proven that allergic respiratory diseases can cause obesity in adolescents and have not identified any difference in physical activity (PA) and sedentary behavior (SB) between adolescents with allergic respiratory diseases and those who had any other diseases. Moreover, a review article based on some studies of adolescents with asthma revealed the temporal association between asthma and overweight in youth supported the mediation of two variables (i.e., asthma and overweight) by reduced PA [17]. However, the study investigated only one factor (i.e., asthma) in the allergic respiratory diseases, and it may be difficult to conclude that allergic respiratory diseases can cause obesity because previous studies did not measure major health-related variables such as SB.

The most common treatment for allergic respiratory diseases is pharmacological treatment [18,19]; however, most of the medications alleviate allergic symptoms temporarily that may recur or worsen if they discontinue them, and using drugs or steroids can lead to having undesired side effects (i.e., stunted growth, damage to the quality of life, and hypothyroidism) during adolescence [20]. Therefore, the International Asthma and Allergic Rhinitis and its Impact on Asthma (ARIA) have recommended participation in regular PA to increase immunity against allergy-causing factors [21,22]. Likewise, guidelines from other countries have also encouraged to participation in regular moderate to vigorous-intensity physical activity (MVPA) for adolescents with asthma and allergic rhinitis [23,24,25,26].

Despite many guidelines recommending regular PA, adolescents who have allergic respiratory diseases tend to spend most of their time in sedentary activities [27]. Some investigators have proposed adolescents with allergic respiratory diseases may be discouraged from participating in PA and are more likely to be sedentary [28,29]. In addition, the association between asthma or allergic rhinitis and obesity has not yet been elucidated. The results on PA and body mass index (BMI) levels affecting adolescents with allergic respiratory diseases were controversial in previous studies [30,31]. Therefore, the aim of the cross-sectional study was to investigate the impacts of allergic respiratory diseases on PA, SB, and BMI by matching age and gender with those adolescents without allergic respiratory diseases.

## 2. Materials and Methods 

### 2.1. Design

The Korea Youth Risk Behavior Web-based Survey (KYRBWS) is an annual project conducted by the Korea Centers for Disease Control and Prevention (KCDCP) to obtain information on health behaviors (i.e., food and nutrition, mental health, and chronic diseases) of Korean adolescents. The 2019 KYRBWS was conducted by an anonymous online survey of Korean adolescents aged from 12 to 18 years (7th–12th grade) on 60,100 adolescents and a total of 57,303 adolescents participated in this cross-sectional study (95.3% participation rate). Among adolescents who responded to the survey, the study consisted of an asthma group and an allergic rhinitis group based on an asthma-related question and an allergic rhinitis-related question, respectively, and the general group was formed by matching age and gender with the previous two groups’ adolescents (i.e., asthma and allergic rhinitis group).

### 2.2. Participants

A total of 57,303 Korean adolescents responded to the 2019 KYRBWS survey. Among those, weight and height data for calculating BMI were partially missing and excluded (n = 1555). In addition, partially missing PA data and sedentary activity data were excluded (n = 1844). Adolescents with respiratory diseases were selected through asthma and allergic rhinitis-related questions, and all adolescents matched age and gender between adolescents with allergic respiratory diseases and those who had not. Adolescents in the asthma group checked on an asthma-related question (i.e., have you been diagnosed with asthma in the last 12 months?) as ‘yes’ (n = 259) and the allergic rhinitis group’s adolescents also checked on an allergic rhinitis-related question (i.e., have you been diagnosed with allergic rhinitis in the last 12 months?) as ‘yes’ (n = 259). In contrast, adolescents in the general group responded to the questions like above as ‘no’ (n = 259) and we randomly selected adolescents and carefully matched the age and gender for the general group from the remaining adolescents.

### 2.3. Ethics Approval

Before participating in the survey, students were thoroughly received about the survey’s purpose and process from the trained teacher and written informed consent themselves. The Korean Youth Risk Behavior Web-based Survey (KYRBS) was performed by the government office in South Korea and it was approved by the institutional review board of the Korean Centers for Disease Control and Prevention. The utilization of statistical data obtained from KYRBS was approved by the national legislation (National Health Promotion Act). The present study used the available raw data, so the study was waived by the institutional review board of Kyung Hee University.

### 2.4. Body Mass Index

The most practical method of measuring the level of obesity is body mass index (BMI) and it was calculated by dividing the weight (kg) by the square of the height (m^2^). Weight was divided into four categories in this study: underweight, normal, overweight and obese according to clinical meaning based on BMI. The method of converting BMI into four clinical categories has some differences in criteria cut-offs depending on the distribution of weight in the western and eastern populations. The standard criteria cut-offs for the Asian-Pacific region in BMI terms are underweight (<18.5 kg/m^2^), normal weight (18.5–22.9 kg/m^2^), overweight (23.0–24.9 kg/m^2^), and obesity (>25.0 kg/m^2^) [32]. The present study also utilized the standard BMI criteria of the Asia Pacific region. 

### 2.5. Self-Report Physical Activity

The Global Physical Activity Questionnaire (GPAQ) translated to a Korean-language version was applied and it has been proven with high reliability and validity [33]. The questions surveyed the different intensities of PA (i.e., moderate and vigorous) during a week. The questionnaire has been validated in several different countries and the data were analyzed by applying the procedures presented in the Global Health Organization’s GPAQ Analysis Guideline. PA was calculated by “metabolic equivalent task (MET) level × minutes × number of days per week” for each intensity, with 4.0 METs for moderate physical activities and 8.0 METs for vigorous physical activities. Furthermore, to determine whether PA affects the BMI levels, the average participating time of PA for adolescents recommended by WHO was calculated as METs (4.0 METs × 60 min × 7 Days) and divided into two categories: above-standard PA and below-standard PA [34].

### 2.6. Self-Report Sedentary Behavior

SB was determined by asking the participants how many hours they sit for study and leisure time during a week. SB for the study was calculated by sitting time in their house, school, and private institute (included using a computer for studying and watching educational broadcasting). The SB for leisure was calculated by time not related to studying (i.e., watching TV, playing video, and online games). According to the Global Health Organization’s GPAQ Analysis Guideline, SB was calculated by “MET level × minutes × number of days per week” for each intensity, and 1.0 METs for sitting time. In addition, to demonstrate whether SB affects the level of BMI, the average sitting time of adolescents presented by Australia’s Physical Activity and Sedentary Behavior Guidelines was calculated as METs (1.0 METs × 120 min × 7 Days) and divided into two categories: above-standard SB and below-standard SB [35].

### 2.7. Data Analysis

The demographic information (i.e., gender, age, and anthropometrics) of all participants who were divided into three different groups (i.e., general, asthma, and allergic rhinitis group) was examined using descriptive statistics in SPSS 25.0 version (IBM, Chicago, IL, USA). All participants’ anthropometrics and personal information were compared between different groups with a series of analysis of variance (ANOVA) with Bonferroni post-hoc analysis. The *p*-value was used to explore whether the association between variables (i.e., demographic and body anthropometrics) and each group. In addition, multinomial logistic regression was utilized to investigate how respiratory diseases affect weight status expressed in terms of BMI. The BMI levels were included in the multinomial logistic regression analysis as independent variables and the three different groups, MVPA, and SB were used as a dependent variable. Results from this analysis are presented as odds ratios (OR) with 95% confidence interval (CI) and statistical significance set by *p* < 0.05.

## 3. Results

Table 1 presents the demographic characteristics of adolescents using descriptive statistics. Participants’ demographic characteristics (i.e., gender and age) and anthropometric measurements (i.e., height, weight, and BMI) are summarized as the frequency and proportions. The sample in the present study included 777 adolescents and the mean of ages was 14.73 years; males were 61% and females were 39% in each group (i.e., general, asthma, and allergic rhinitis group). The adolescents’ characteristics (i.e., gender and age) indicated no significant differences among the three different groups because age and gender were matched for each group. With regard to the anthropometry, height also showed no significant differences among the three groups (*p* = 0.75); however, weight and BMI presented differences among those groups, *p* = 0.001 and *p* = 0.001, respectively.

Table 2 presents the multinomial logistic regression model to examine whether the factors of respiratory disease, MVPA, and SB were associated with the weight classes expressed in terms of BMI (>25.0, 23.0–24.9, and <18.5 kg/m^2^) in adolescents. Some variables (i.e., below-standard MVPA and below-standard SB) do not show an association with obesity (BMI > 25.0 kg/m^2^), however, age (*p* = 0.007; OR = 1.171; CI = 1.044–1.313), asthma (*p* < 0.001; OR = 8.095; CI = 4.109–15.947), and allergic rhinitis (*p* < 0.001; OR = 10.247; CI = 4.977–21.096) were observed to be strong risk factors for predicting obesity in adolescents (BMI >25.0 kg/m^2^). Furthermore, asthma (*p* < 0.001; OR = 7.081; CI = 3.170–15.819) and allergic rhinitis (*p* < 0.001; OR = 12.100; CI = 5.212–28.089) had significant impacts on overweight (BMI 23.0–24.9 kg/m^2^), but age, MVPA, and SB also were not the relevant risk factors for overweight in terms of BMI. In contrast, only an age variable (*p* < 0.001; OR = 0.746; CI = 0.657–0.847) was a significant factor in predicting underweight (BMI < 18.5 kg/m^2^).

**Figure 1:**
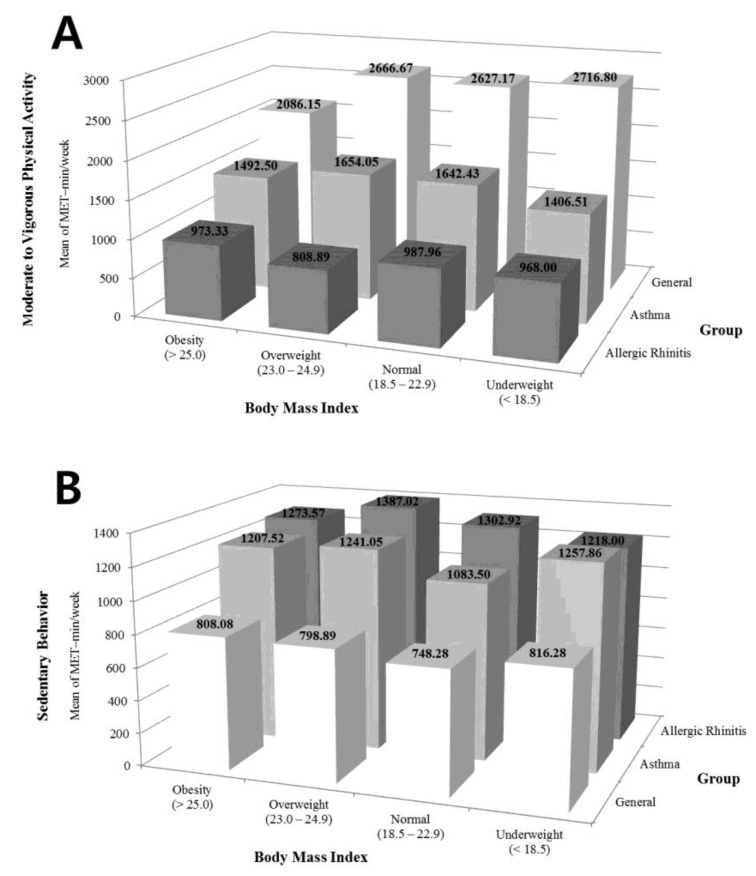
The comparisons of body mass index (BMI) and participating time in moderate to vigorous physical activity (**A**) and sedentary behavior (**B**) during a week among healthy, asthma, and allergic rhinitis group.

Specifically, Figure 1 revealed the combined analyses of changes in MVPA (Figure 1A) and SB (Figure 1B) between adolescents with or without allergic respiratory diseases. The general group’s adolescents in all BMI levels indicated the highest participation in MVPA; an average of 2086.15 MET-minute/week in obesity (>25.0 kg/m^2^), 2666.67 MET-minute/week in overweight (23.0–24.9 kg/m^2^), 2627.17 METs in normal (18.5–22.9 kg/m^2^), and 2716.80 MET-minute/week in underweight (<18.5 kg/m^2^). However, adolescents with asthma have much lower MVPA than those in the general group (obesity: 1492.50 MET-minute/week, overweight: 1654.05 MET-minute/week, normal: 1642.43 MET-minute/week, underweight: 1406.51 MET-minute/week). Moreover, the allergic rhinitis group’s adolescents spent the less time participating in MVPA among the three different groups (obesity: 977.33 MET-minute/week, overweight: 808.89 MET-minute/week, normal: 987.96 MET-minute/week, underweight: 968.00 MET-minute/week), and asthma + obesity group had twice as much less time to participate in PA as the general group, and even the allergic rhinitis + overweight group was three times less than the general group. No interaction was found between groups and BMI levels (*p* = 0.554), however, MVPA (i.e., MET-minute/week) shows the significant differences within the three groups regardless of BMI levels (*p* = 0.001). 

Conversely, examinations according to nine combinations of changes in SB (Figure 1B) illustrate the opposite results compared with the outcomes of MVPA. In the general group, adolescents with obesity or overweight group participated in SB averaged 808.08 and 798.89 MET-minute during a week. However, the adolescents with asthma in overweight or obesity have almost doubled the number of MET-minute for SB (overweight: 1241.05 MET-minute/week, obesity: 1207.52 MET-minute/week) compared to adolescents of the general group. Thus, although there were no huge differences when combined with obesity and overweight in the asthma group, the allergic rhinitis group’s adolescents in obesity or overweight spent more time in SB (obesity: 1273.57 MET-minute/week, overweight: 1387.02 MET-minute/week). No interaction was found between groups and BMI levels (*p* = 0.591), however, SB (i.e., MET-minute/week) shows the significant differences within three groups regardless of BMI levels (*p* = 0.001).

## 4. Discussion

Allergic respiratory diseases in adolescence are not life-threatening, but these are likely to persist chronically into adulthood, and more than half of the adolescents with allergic respiratory disease may experience a recurrence in adulthood [36]. Moreover, adolescents with allergic respiratory disease have suffered from severe interpersonal difficulties because of restricted daily physical activities [37]. Despite this, studies related to allergic respiratory diseases are not yet fully understood and needed further investigation as to what other factors (i.e., PA, SB, and BMI) may be associated with allergic respiratory diseases. 

The present study divided participants into three groups (general, asthma, and allergic rhinitis), and compared anthropometry (i.e., gender, age, height, and weight) as well as the differences of MVPA, SB, and the levels of BMI with the age- and gender-matched groups. The results showed that the weight and BMI were significantly higher in the disease groups (asthma and allergic rhinitis) compared to the general group, but no difference between asthma and allergic rhinitis was found in the study. Although it is difficult to determine the direct relations between respiratory diseases and BMI levels, our findings demonstrated adolescents with asthma and allergic rhinitis groups were heavier and higher BMI than the general group.

Furthermore, the findings from multinomial logistic regression revealed multiple factors played a significant role in affecting the adolescents’ weight status, perhaps including age. Age had significant impacts on obesity (*p* = 0.007, OR = 1.171) and underweight (*p* = 0.000, OR = 0.746), so with increasing age, the tendency to become obese has increased, while the number of underweight adolescents has gradually decreased. This pattern may be explained by the general phenomenon of Korean society in which most adolescents spend their majority time studying. The outcomes by Statistics Korea and the Ministry of Gender Equality and Family [38] also indicated that participating in regular physical activities have been gradually decreased as their grade increased during adolescence (elementary school students: 75.3%, middle school students: 51.4%, and high school students: 40.1%). Furthermore, they have significantly less sleeping time due to their academic pressures. A recent study also pointed out that adolescents in South Korea showed the highest level of physical inactivity out of 146 countries [39]. Therefore, immediate action plans would need to implement public health initiatives in schools to make adolescents be more active.

One interesting result of the study was a strong impact of allergic respiratory diseases on different BMI levels because adolescents with asthma or allergic rhinitis were more likely to become obese than general adolescents about eight times and 10 times higher, respectively. To be specific, compared to the general group, the probability of the asthma group becoming overweight was 7 times higher, and becoming obese was 8 times higher. An even worse scenario would be adolescents in the allergic rhinitis group were more likely to become obese and overweight about 10 to 12 times greater compared to the general adolescents. Therefore, adolescents with ongoing asthma, or allergic rhinitis symptoms, were more likely to have the inevitability of further weight gain. Although many factors contribute to the fact that adolescents in the allergic rhinitis group are more likely to be obese than those in the asthma group, one possible factor is the bothersome symptoms of rhinitis (i.e., rhinorrhea, sneezing, postnasal drip, daytime fatigue, and sleep disturbance) [40]. These bothersome symptoms affect not only the limitation of daily PA but also the increased SB and the decreased quality of life, so adolescents with allergic rhinitis are much more vulnerable to stress and discouraged efforts to participate in physical activities and more likely to become obese and sedentary than adolescents with asthma [41,42]. These findings indicated that allergic respiratory diseases (i.e., asthma and allergic rhinitis) can be one of the main causal factors for obesity. As such, public health practitioners should pay more attention to allergic respiratory diseases that occur frequently in adolescence, and the results of this study suggest that specific PA guidelines should be provided for adolescents with allergic respiratory diseases to prevent obesity and maintain a normal weight.

Specifically, the MET-minutes/week for MVPA was the highest in the general group than the other two groups (asthma and allergic rhinitis). While the asthma group participated in two times lower MVPA, the allergic rhinitis group was performed three to four times lower MVPA compared to the general group. Similar results were observed for SB where asthma and allergic rhinitis groups spent approximately two times higher for SB than the general group. One of the reasons for decreased MVPA and increased SB was that adolescents who were diagnosed with allergic respiratory diseases might have concerns about exacerbations of their diseases while participating in PA. Although adolescents with allergic respiratory diseases are reluctant to participate in PA, it is important to know that PA or exercise is not a risk factor for asthma and participating in vigorous PA positively affected individuals with allergic respiratory disorders [43,44]. In addition, one of the studies also found that individuals who participated in regular PA (i.e., MVPA) do not worsen the allergic rhinitis symptoms [45] and factors such as regular participation in MVPA are important to consider because it improves their pulmonary function [46]. Therefore, we might speculate that the future PA recommendation guideline for adolescents with the allergic respiratory disease would be considered its severity (i.e., mild and moderate-severe) and persistence (i.e., intermittent and persistent) [27]. Moreover, most adolescents have a mild symptom of asthma and allergic rhinitis [47] and PA is the critically important factor to prevent adolescents with allergic respiratory diseases from being exposed to other diseases obtained from obesity. 

The present study had several positive strengths. To the best of our knowledge, no study to date has examined the association between MVPA, SB, and BMI and respiratory diseases in adolescents with age- and gender-matched population. In addition, the main findings of the study add to the existing literature on the association between allergic respiratory diseases and adolescents’ weight status and also provide new insights regarding the factors contributing the respiratory diseases such as MVPA and SB. However, some limitations still exist in the current study. This study has restricted some factors that can affect obesity by matching the age and gender of all participants. However, further studies would examine the association between adolescents with allergic respiratory diseases and obesity in the longitudinal follow-up study. In addition, we suggest that future studies will be able to investigate various factors (i.e., race, mental health, parental educational levels, or income) that lead to obesity in adolescents with allergic respiratory diseases. In addition, the level of PA was measured by self-reported questionnaires instead of utilizing objective measures (i.e., accelerometer), which may cause an under- or over-estimation of the associations between PA and other health outcomes. Therefore, it is necessary to apply objective methods such as accelerometers to measure more valid and reliable PA. Participants in the present study were limited to Korean adolescents, so additional studies need to demonstrate more evidence with various ethnicity and adequate sample size to examine the association between general adolescents and BMI status. 

## 5. Conclusions

The present study supports that adolescents with allergic respiratory diseases were more likely to become obese than normal adolescents, especially adolescents with allergic rhinitis are more vulnerable to maintaining normal BMI than adolescents with asthma. To alleviate allergic respiratory diseases, pharmacological treatments (i.e., using drugs or steroids) are the most common medications, however, based on the results of this study, increased PA and reduced SB can be one of the effective treatments for allergic respiratory diseases in adolescents.

## Figures and Tables

**Table 1:** Characteristics and anthropometrics for participants in three groups.

Variables		General (N = 259)		Asthma (N = 259)		Allergic Rhinitis (N = 259)	
		No. (%)	Mean ± SD	No. (%)	Mean ± SD	No. (%)	Mean ± SD
Gender	Male	158 (61.00)		158 (61.00)		158 (61.00)	
	Female	101 (39.00)		101 (39.00)		101 (39.00)	
Age (year)	12 yr	29 (11.20)		29 (11.20)		29 (11.20)	
	13 yr	53 (20.46)		53 (20.46)		53 (20.46)	
	14 yr	42 (16.22)		42 (16.22)		42 (16.22)	
	15 yr	42 (16.22)		42 (16.22)		42 (16.22)	
	16 yr	37 (14.29)		37 (14.29)		37 (14.29)	
	17 yr	39 (15.05)		39 (15.05)		39 (15.05)	
	18 yr	17 (6.56)		17 (6.56)		17 (6.56)	
Height (cm)	Male	158 (61.00)	166.17 ± 6.37	158 (61.00)	164.09 ± 9.13	158 (61.00)	165.84 ± 6.06
	Female	101 (39.00)	158.79 ± 6.31	101 (39.00)	158.94 ± 5.55	101 (39.00)	157.85 ± 5.05
Weight (kg)	Male	158 (61.00)	55.92 ± 9.04	158 (61.00)	59.22 ± 13.92 ***	158 (61.00)	61.28 ±10.60 ***
	Female	101 (39.00)	49.09 ± 7.40	101 (39.00)	53.51 ± 10.35 ***	101 (39.00)	52.79 ± 9.28 ***
BMI (kg/m^2^)	Male	158 (61.00)	20.18 ± 2.52	158 (61.00)	21.87 ± 4.26 ***	158 (61.00)	22.19 ± 3.07 ***
	Female	101 (39.00)	19.42 ± 2.36	101 (39.00)	21.11 ± 3.45 ***	101 (39.00)	21.22 ± 3.85 ***

SD: standard deviation, *** *p* < 0.001.

**Table 2:** Summary of multinomial logistic regression analysis.

Variable		Obesity (>25.0 kg/m^2^)			Overweight (23.0–24.9 kg/m^2^)			Underweight (<18.5 kg/m^2^)		
		*β*	S.E	OR (95% CI)	*β*	S.E	OR (95% CI)	*β*	S.E	OR (95% CI)
Age		0.158	0.059	1.171 ** (1.044–1.313)	0.054	0.067	1.056 (0.926–1.203)	−0.293	0.065	0.746 *** (0.657–0.847)
Groups	General			1.000 (1.000–1.000)			1.000 (1.000–1.000)			1.000 (1.000–1.000)
	Asthma	2.091	0.346	8.095 *** (4.109–15.947)	1.957	0.410	7.081 *** (3.170–15.819)	0.137	0.267	1.147 (0.680–1.935)
	Allergic Rhinitis	2.327	0.368	10.247 *** (4.977–21.096)	2.493	0.430	12.100 *** (5.212–28.089)	−0.208	0.316	0.813 (0.437–1.510)
Physical Activity	Above-Standard MVPA			1.000 (1.000–1.000)			1.000 (1.000–1.000)			1.000 (1.000–1.000)
	Below-Standard MVPA	−0.009	0.244	0.991 (0.615–1.599)	−0.221	0.276	0.802 (0.467–1.378)	0.137	0.246	1.146 (0.708–1.856)
Sedentary Behavior	Above-Standard SB			1.000 (1.000–1.000)			1.000 (1.000–1.000)			1.000 (1.000–1.000)
	Below-Standard SB	0.046	0.235	1.047 (0.660–1.660)	0.083	0.270	1.086 (0.640–1.843)	0.372	0.232	1.451 (0.922–2.285)

*** *p* < 0.001, ** *p* < 0.01, OR: odds ratio, CI: confidence interval, MVPA: moderate-vigorous intensity physical activity, SB: sedentary behavior; standard MVPA: means participating in MVPA more than 1680 metabolic equivalent tasks (METs) during a week (60 min × 7 days × 4.0 METs), standard SB: means sitting for study and leisure more than 840 METs during a week (120 min × 7 days × 1.0 METs).
